# Equity Across Religious Identity: Assessing Student Attitudes and Experiences with the Medical School Religious Holiday Policy

**DOI:** 10.1089/heq.2024.0066

**Published:** 2024-08-08

**Authors:** Sarah Battiston, Emily Otiso, Dustyn Levenson, Haniyeh Zamani, Ijeoma Nnodim Opara

**Affiliations:** Wayne State School of Medicine, Detroit, Michigan, USA.

**Keywords:** religious, equity, policy

## Abstract

**Background:**

Wayne State University School of Medicine (WSUSOM) is the largest single-campus medical school located in a diverse community. WSUSOM’s religious holiday policy guarantees time off for observance of one religious holiday. For all other religious holidays, students must request for time off. The current policy lacks specific guidelines to ensure equity across religious identities when granting time off. Religious and spiritual practice can enhance wellness. Therefore, assessing the equity of the current policy is crucial to ensuring equitable access to wellness.

**Objective:**

This project aims to assess students’ attitudes and experiences with the current religious holiday policy at WSUSOM and compare experiences across religious identities.

**Methods:**

A 17-question Qualtrics survey was emailed to all WSUSOM students. Survey questions included demographics, experiences with the current policy, and Likert scales to assess attitudes. Data was analyzed holistically and assessed for variation among religious identities using chi-squared analysis.

**Results:**

Analysis included 156 surveys: 27.5% of students reported difficulties getting their religious holiday off, and 9.8% were denied a religious holiday, Muslims being the most impacted (*p* < 0.01). Muslim identifying students (75%) reported the highest incidence of completing additional work to receive an absence; 35.6% of students agreed that the current policy caused mental distress and majority of those being Muslim students (*p* < 0.01).

**Conclusions:**

The current policy has caused difficulty for many students and has disproportionately impacted students from minority religions (especially Islam), exposing the need for a new policy.

## Background

There is a growing recognition of the importance of diversity in medicine, with significant efforts focused on increasing diversity in the medical student population.^1^ The Liaison Committee on Medical Education (LCME) and other major medical organizations have called for the implementation of various antidiscrimination policies to support a diverse student body, including protection from discrimination against religious identity.^2–4^

The ability to support a diverse student body is crucial to student well-being. Religion and spirituality can be essential in coping with stress and overall wellness in physicians, residents, and medical students.^5–9^ However, medical students tend to lack time to use these protective practices due to the demanding nature of medical school and the lack of administrative policies to protect time for religious and spiritual practice.^10^ Therefore, institutions must implement policies to protect their students’ religious and spiritual practices and promote an equitable environment for all religious/spiritual identities.

Wayne State University School of Medicine (WSUSOM) is an urban medical school in Detroit, Michigan. Part of the WSUSOM mission includes educating a diverse student body within a culture of inclusion, and indeed WSUSOM is ranked as the 28th most diverse institution among United States (US) medical schools.^11,12^ The most recently admitted WSUSOM class (2026) demographics consisted of 50% Caucasian, 32% Asian, 8% Black, 7% Mixed Race identifying students, and 3% other.^13^ Given the diversity at WSUSOM, it is crucial to support students of all identities, including those holding minority religious identities.

The WSUSOM Religious Holiday Policy guarantees time off for observance of one preapproved religious holiday recognized on the academic calendar (Christmas), during which the university is closed for winter break. All students regardless of religious identity, therefore, get this Christian holiday off. Other nonreligious holidays are similarly preapproved when they align with university closure (i.e., Thanksgiving). For all other religious holidays, students are expected to attend scheduled academic events, and must request an excused absence. The policy guides students to contact their counselor, who then works with the clerkship/elective director to determine if the request is considered appropriate and what actions must be completed by the student to remediate time off. Of note, many clinical duties for students extend into weekend days, meaning any religious holiday that lands on a weekend must also be requested off, including Easter. Therefore, all students, regardless of religious identity are at subject to this policy.^14^

***Policy:*** *“Requests for time away from clerkships and electives must be submitted in writing to the student’s counselor as soon as possible upon knowing of the need for an excuse. The student’s counselor will work with the student to contact the Clerkship/Elective Director to request the time off if the request is considered appropriate. The counselor and student will work with the Clerkship/Elective Director to determine how/if the time can be made up. Excused absences may not be granted by the Clerkship/Elective Director if this policy is not followed.”^14^(p166)*

No specific guidelines exist within the policy detailing what is considered to be an appropriate request or how to dictate make-up time and assignments uniformly and equitably. The lack of clear guidelines within the policy leaves these decisions up to the subjectivity of the counselor/director and, therefore, strongly open to bias. Although it is unknown how this policy impacts students across religious identities, there is substantial evidence demonstrating that those holding racial or religious minority identities face heightened bias and discrimination without the protection of uniform policies.^15–17^ Therefore, there is an urgent need to elucidate the ways by which religious policies perpetuate the marginalization of those holding minority identities within the context of medical education. Herein, the objective of the current study was to assess students’ attitudes and experiences with the current religious holiday policy at WSUSOM and compare these experiences across religious identities.

## Methods

### Study Design

The researchers submitted this cross-sectional study to the Institutional Review Board (IRB) at WSUSOM. The IRB granted an exemption owing to the classification of this study as minimal risk. Researchers chose a survey as the data collection method because it allowed a large number of students to report their subjective experiences and attitudes toward the policy in an anonymous format.^18^ The study’s target population was all students attending WSUSOM in 2022. Students were recruited to the study using a class-wide email list, and participation in the survey was completely voluntary and anonymous. Overall schoolwide population size and demographics were determined by records obtained from WSUSOM’s records office.^19^

### Instrument Development

We conducted a systematic literature review of PubMed to identify prior studies regarding institutional religious holiday policies or studies that evaluated students’ experiences with or attitudes toward other institutional policies. Many studies focused on the role of religiosity on the well-being of medical students.^5–9^ However, to our knowledge, no studies exist that evaluate experiences/attitudes with a religious holiday policy or any other institutional policies. Therefore, student researchers created survey questions in conjunction with an expert in the field, who served as the Vice Dean of Diversity. A pilot test was distributed to six students with diverse religious backgrounds, including Christian, Muslim, Jewish, and Agonistic identities. Researchers held cognitive interviews with each of these six students. These one-on-one pilot sessions covered verbal feedback for each question, comprehensibility, and overall ease and use of the survey.

The final survey was created using Qualtrics and contained 17 questions. The survey contained three sections: demographics, experiences with the current policy, and attitudes/beliefs about the current policy. The demographics evaluated included year in medical school, gender, religious, and racial identity. The experiences section of the survey used seven questions of various styles to obtain subjective data on students’ experiences, including multiple-choice, choose all-that-apply, and fill-in-the-blank. The questions in this section covered difficulties obtaining an excused absence for a religious holiday, denial of an excused absence, and extra work required to obtain the approval for the absence. The attitudes/beliefs section contained six statements that students rated on a 5-point Likert scale (5= strongly agree, 1= strongly disagree).^20^ These statements included themes of mental distress caused by the policy, fairness toward minority students, opinions on revision and the need for a new policy, and the importance of institutional support for religious/spiritual identity. The last survey question was an optional fill-in-the-blank question that allowed students to share anything they felt necessary for the researchers to know regarding their experiences. The complete survey may be found in the Supplementary Appendix S1.

### Data Collection Methods

Researchers emailed the Qualtrics survey to all WSUSOM students using a class-wide email list on September 21, 2022. The email included a link to the survey, and participation was voluntary. The survey closed over two weeks later on October 7, 2022, and two reminders were sent to students in the interim to promote participation and reduce nonresponse bias. The survey was anonymous and did not obtain identifying information.

### Statistical Analysis

The initial analysis investigated the distribution of responses across the overall student body. After initial analysis, students were grouped based on religious identity and survey responses were compared. Religions with fewer than three responses were grouped into a single category entitled “other.” The religious identities that were included were Christian, Muslim, Hindu, Jewish, Atheist/Agnostic, and other. Researchers then assessed variations attributed to religious identity using chi-squared tests (SPSS for Windows, Version 18, Chicago, IL, USA). A *p* value of <0.05 was considered statistically significant. Exclusion criteria included surveys that did not progress past demographics (36% complete).

## Results

We obtained 331 surveys, 170 of which did not progress past demographics and were excluded from data analysis. An additional five surveys were excluded owing to respondents not listing a religious identity or responding with more than one. A total of 156 (47.1%) surveys were analyzed, and the response rate was 13.4%.

### Demographics

The demographics of the survey respondents and study population are displayed in Table 1. The majority of respondents identified as Christian (41.0%), followed by Muslim (23.1%). The gender and racial composition of respondents were reflective of the medical school body composition (Table 1). However, a discrepancy was identified in our composition of racial identity when compared with WSUSOM’s reported demographics, which is complicated by a lack of consistency among demographic sampling parameters.^19^ Our study allowed students to write-in their race and included a “Middle Eastern” category, whereas the school did not include this option when obtaining racial identity. Furthermore, the WSUSOM report of student demographics did not include students’ religious identity, preventing comparison of religious distribution between our study and WSUSOM as a whole.

**Table 1.** Demographics of Survey Respondents and Study Population and Covariate Analysis

	Demographics		Atheist/Agnostic	Christian	Muslim	Hindu	Jewish	Other	*p* value
	Survey Respondents	WSUSOM Students							
Gender Identity									0.65
Female	60.3%	51.3%	14	38	21	9	9	3	
Male	39.1%	48.6%	8	25	15	7	4	2	
Nonbinary	0.6%	0%	0	1	0	0	0	0	
Year^a^									0.66
M1	28.2%	26.0%	8	17	9	7	1	2	
M2	19.2%	28.0%	4	12	5	4	4	1	
M3	32.7%	22.8%	6	24	15	1	4	1	
M4	17.9%	23.2%	4	10	5	4	4	1	
LOA	1.9%	—	0	1	2	0	0	0	
Racial identity^b^									0.01
White	48.7%	47.5%	18	43	3	0	11	1	
Black/African American	5.1%	8.2%	1	5	1	1	0	0	
African	0.6%	—	0	0	1	0	0	0	
Middle Eastern	13.5%	—	0	4	16	0	1	0	
Asian	25.0%	22.8%	2	6	13	15	0	3	
Hispanic	1.3%	9.0%	0	1	0	0	0	1	
Mixed Race	5.1%	3.5%	0	5	2	0	1	0	
Decline	0.6%	5.2%	1	0	0	0	0	0	
Other	—	4.0%							
Total	N*n* = 156	*N* = 1,173	22	64	36	16	13	5	

^a^ School reported records do not include student on leave of absence (LOA), due to LOA students’ ability to enter back into the curriculum at multiple points during the semester. Therefore, the year’s total is 1,170.

^b^ School records had differing subgroupings for race than included in the survey

Covariates of interest included year, gender, and racial identity. Chi-squared analyses revealed that the *p* values of year and gender were not significant (Table 1). However, the *p* value for racial identity was significant (*p* < 0.01, Table 1). Although race was not controlled for within this study, we recognize that race is a sociopolitical construct and cannot disregard the role of systemic racism on our results.^21^

### Experiences

**FIG. 1.**
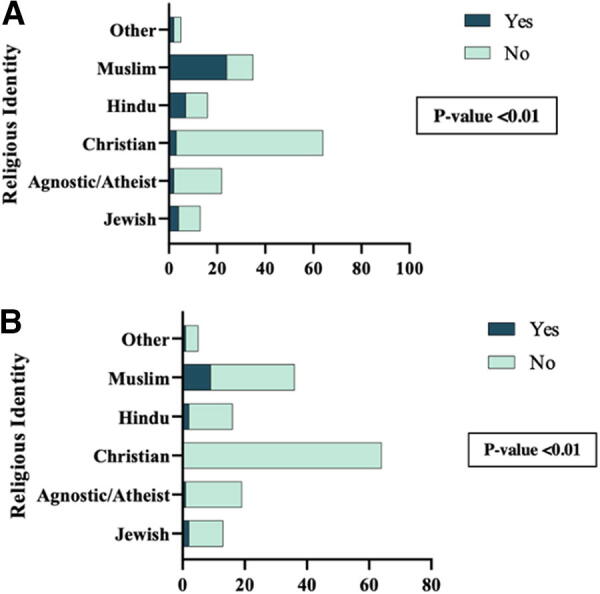
Difficulties with the current policy and denial of excused absence across religious identity. **(A)** Have you had difficulties getting an excused absence for a holiday under your personal religious identity? **(B)** Have you been denied an excused absence when requesting it for a religious holiday?

The survey assessed students’ self-reported difficulty obtaining an excused absence and denial of an excused absence for their religious holiday. Although only 27.5% of students reported difficulties when requesting an excused absence for their religious holiday, we found that students who identified as Muslim, as well as by Hindu, were more likely to have difficulties compared with the other identities (*p* < 0.01; Fig. 1a). Students who reported difficulties obtaining an excused absence were asked to specify those difficulties and were able to choose all that applied. The majority of students (52.3%) reported issues with receiving approval from the advisor/clerkship director/course director. Others reported difficulties with make-up assignments (50.0%), finding/understanding current policy (42.8%), and contacting their advisor (33.3%). When asked if the student was denied an excused absence (Fig. 1b), 9.8% of students reported that they had experienced being denied. Again, students who identified as Muslim had a significantly higher frequency of being denied than other religious identities (*p* < 0.01). None of the Christian identifying respondents reported being denied a religious holiday.

**FIG. 2.**
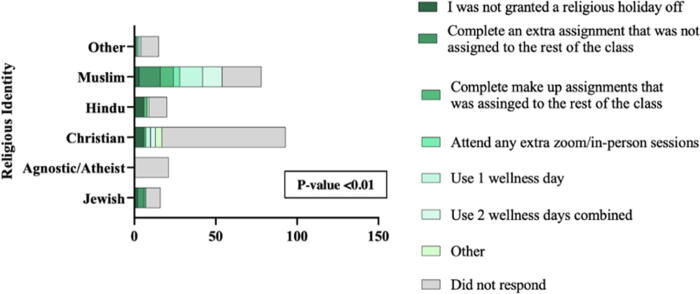
If you were granted an absence for a religious holiday, did you have to do any of the following (select all that apply):

Students who were granted an excused absence were asked if they had to complete any extra work to receive the absence (Fig. 2). There was a statistically significant difference in extra work among religious identities, with Muslim and Jewish identifying students having the highest likelihood of completing extra work (*p* < 0.01). Among all of the respondents, 18 (11.5%) students had to complete an assignment that was not assigned to the rest of the class, 13 (8.3%) completed an assignment that was assigned to their class, and 4 (2.6%) students were required to attend an extra in-person or online class. Lastly, 18 (11.5%) students were required to use one wellness day* and 16 (10.2%) used two wellness days.

*Wellness days are a half-day allotted to clerkship students to use at will for wellness-related activities. Wellness days are limited by the length of clerkship rotation: 1 half-day per 4- and 6-week long rotations, and 2 half days per 12-week rotation.^22^

### Attitudes

**FIG. 3.**
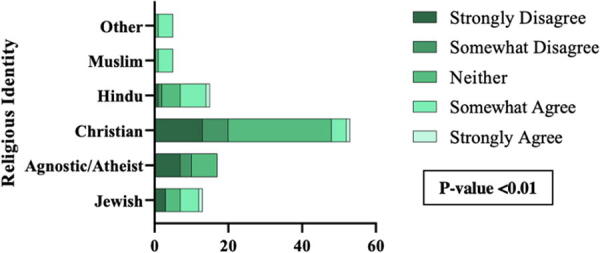
The current religious holiday policy or any difficulties faced in trying to request exemption for a religious holiday caused me distress/mental health concerns.

The survey asked students to rank statements on a 5-point Likert scale (1 = strongly disagree to 5 = strongly agree). The Likert scale statements focused on the current religious holiday policy, a potential future policy, and the importance of institutional support of religious identity. About 35% of students somewhat agree or strongly agree that the current religious holiday policy or difficulties requesting an exemption caused them distress/mental health concerns. Analyses showed statistically significant associations between identifying as Muslim and agreeing with this statement (*p* < 0.01; Fig. 3). Hindu and Jewish identifying students also had a higher likelihood of agreeing with this statement. The remaining Likert-scale questions are outlined in Table 2. Results showed that most students agreed that the policy needs revisions with clear guidelines and that their institution must support their religious beliefs. Lastly, most students agreed that a new policy is needed to create an inclusive environment and disagreed that the policy is equitable to students of all religious backgrounds.

**Table 2.** Likert-Scale Questions Assessing Student’s Attitudes to Various Statements Regarding the Current and Possible Future Religious Holiday Policy

	Strongly disagree	Somewhat disagree	Neutral/neither agree or disagree	Somewhat agree	Strongly agree
a) The current religious holiday policy needs to be revised with clear guidelines for requesting off a religious holiday	6 (4.5%)	2 (1.5%)	45 (34.1%)	41 (31.1%)	38 (28.8%)
b) The current religious holiday policy is fair toward students of minority religions	31 (23.5%)	33 (25.0%)	52 (39.4%)	9 (6.8%)	7 (5.3%)
c) A new religious holiday policy would create a more inclusive environment	1 (0.7%)	0 (0%)	35 (26.5%)	40 (30.3%)	56 (42.4%)
d) A new religious holiday policy that would not use wellness days would positively impact my mental health	1 (0.7%)	4 (3.0%)	34 (25.8%)	29 (22.0%)	64 (48.5%)
e) It is important to me that institutions support my religious beliefs by allowing me time off for my holidays	2 (1.5%)	3 (2.3%)	22 (16.7%)	25 (18.9%)	80 (60.6%)

*N* = 132.

### Student Voice

Researchers allotted students the space to write any concerns at the bottom of the survey. Many student voices echoed similar themes of an inequitable policy.

*“The current policy says students are excused by default on university-approved holidays. However, these holidays cater primarily to majority White and Christian students. Therefore, this policy is inherently discriminatory as it requires minority students to navigate additional bureaucracy to request time off.”*

*“I feel that if Christian students are provided entire weeks off for Christmas, it is unfair for Muslim students to need to work around the schedule, make up work, or miss important learning for the few days we have for Eid. There should be steps to recognize the large population of Muslims at WSUSOM’s campus and not have required activities on the two days of Eid.”*

## Conclusion

This study demonstrated that the Wayne State University School of Medicine’s Religious Holiday Policy has caused difficulties for many students. However, the policy disproportionately impacted students of minority religious identities. Most notably this policy showed clear disparities toward Muslim identifying students. Students identifying as Muslim reported a higher incidence of experiencing difficulties obtaining an excused absence, being denied an absence, having to complete additional assignments to obtain the absence, and reporting higher mental stress. Hindu and Jewish identifying students also reported a high incidence of similar difficulties. Christian identifying students reported less difficulty with the policy when requesting for holidays outside of their guaranteed Christmas holiday.

With a growing call for diversity in medical institutions, institutions must be critically conscious and intentional in supporting their diverse medical student bodies. Perceived religious discrimination is harmful to mental health.^1–4,23^ Medical students who have experienced mistreatment have higher levels of exhaustion, disengagement, career choice regret, and ultimately burnout.^24–26^ Burnout is often a byproduct of depression stemming from sustained distress and is associated with suboptimal patient care, decreased concentration, and an increase in suicidal ideation.^27–29^ Therefore, medical schools must have policies in place to promote religious equity, combat mistreatment, and eliminate discrimination to protect the well-being of their religious minority students. Though this study was focused on a single institution, other institutions’ policies resemble WSUSOM’s religious holiday policy by lacking clearly defined guidelines and leaving the discretion of the excused absence approval and make-up assignments to faculty and other administrators.^30–33^ We hope this initial study lays the groundwork for a larger evaluation of religious holiday policies across multiple institutions.

This study illuminated that the current WSUSOM policy does not equitably grant excused absences for religious holidays and does not ensure an equitable distribution of make-up assignments, classes, and mandatory wellness day use. Importantly, our study demonstrates the unequivocal impact this policy has on students holding minority identities, thus perpetuating discrimination toward socially disadvantaged groups that are already at an increased risk for burnout.

There is a demonstrated need for a new religious holiday policy at WSUSOM that provides definitive language to ensure equity across identities when granting a religious holiday excused absence. Currently, student leadership is creating a more equitable and just policy based on the needs of students, as reflected in this preliminary study. This new policy allocates students a set number of protected excused absences. The current proposal recommends the allocation of three religious holidays excused absence per academic year that students can use at will, removing the need for administrative discretion and minimizing potential bias. The policy specifically states that additional work beyond that of their peers will not be required and specifies that the mandated use of wellness days is not to be suggested or encouraged for religious celebrations unless the student elects to use them in such a manner.

However, in adherence to the educational standards of WSUSOM, students must be conscious of the number of excused absence allowed per block for preclerkship students or per rotation for clerkship and postclerkship students to ensure a balance between absence and educational time. In addition, this new policy will implement a reporting system for policy violations so students can report to administration when violations occur, and resolution can be reached promptly. The goal of the new policy guidelines is providing clear guidelines for students and faculty to use and to reduce bias when granting excused absences. After finalizing and implementing the new policy, researchers intend to repeat a subjective survey-based study on experiences and attitudes, and gather objective data through a tracking system on the use of the religious holiday policy and the number of repeals.

Furthermore, this study raises questions regarding how WSUSOM protects other aspects of religious practice. WSUSOM must take additional steps to evaluate students’ experiences and attitudes with the institution’s support for religious practices, including prayer and religious dress such as hijabs. Currently, little research exists on religious holiday policies within medical schools and the impact of religious holiday policies on medical students. Although this study was completed at a single institution, limiting its generalizability, it raises the larger question regarding how medical institutions nationwide support diverse religious identities among students, residents, and practicing physicians. Researching religious policy content, experiences, and attitudes across institutions is needed to properly evaluate the scope of this problem and assess on which level it should be addressed (i.e., single institution, LCME, etc.). Moreover, assessing various institutional policies would enable institutions to understand what policy language would best ensure equity across various identities. This paper showed statistical significance in self-identified race and religion. Therefore, the next direction of this project is to evaluate the role of intersectionality on variations of experience and attitudes through subgroup analysis.

This study was limited by the use of subjective information obtained from students self-reporting their experiences and attitudes. An additional response bias may exist if those directly impacted by the policy felt more compelled to complete the survey and its small response rate. Lastly, due to no prior publications on this subject, study questions were not adapted from peer-reviewed measurement tools or peer-reviewed publications. Despite these limitations, the data collected provided researchers with a solid understanding of the current policy’s impact on students of varying religious identities.

## Abbreviations Used

LCME: Liaison Committee on Medical Eduation
WSUSOM: Wayne State University School of Medicine
