# Supplementary material for: Equity Across Religious Identity: Assessing Student Attitudes and Experiences with the Medical School Religious Holiday Policy
Source: Health Equity. 2024 Aug 8;8(1):519–26. doi: 10.1089/heq.2024.0066 (PMC11347874; doi:10.1089/heq.2024.0066)
Supplement: Supplementary Appendix S1 [file heq.2024.0066_supplementary_appendix_s1.pdf]

**Survey:**

**Demographics**

Year

- M1
- M2
- M3
- M4
- LOA/gap
- PhD
- Other (please specify)

Gender Identity

- Male
- Female
- Transgender
- Non-binary
- Other (please specify

Racial identity (Fill in the blank)

____________

Religious identity (Fill in the blank)

____________

**Experiences with Current Religious Holiday Policy**

1. Are you aware of/do you know the current excused absence policy for religious holidays?
    a. Yes
    b. No
2. Have you had difficulties getting an excused absence for a holiday under your personal religious identity?
    a. Yes
    b. No
3. If you answered yes to the above question, which of the follow did you have difficulties with? (select all that apply)
    a. Finding/understanding current policy
    b. Contacting advisor/course director/clerkship director
    c. Being approved by advisor/course directory/clerkship director
    d. Making up assignments/work
    e. Other _______
4. Have you been denied of an excused absence when requesting it for a religious holiday
    a. Yes
    b. No

5. If you were granted a religious holiday did you have to do any of the following (select all that apply):
    a. I was not granted a religious holiday off
    b. Complete an extra assignment that was NOT assigned to the rest of the class
    c. Complete make up assignments that was assigned to the rest of the class
    d. Attend any extra zoom/in-person sessions
    e. Use 1 wellness day
    f. Use 2 wellness days combined
    g. Other ____
6. If you selected any of the following from above and want to further explain your situation, please do so below:
    a. ________________________________
7. How many days have you requested off for a religious holiday?
    a. ________________________________

**Attitudes with Current Religious Holiday Policy and Future Directions**

Please rate the following statements below as either strongly disagree, somewhat disagree, neither agree or disagree, somewhat agree, strongly agree

1. The current religious holiday policy or any difficulties faced in trying to request exemption for a religious holiday caused me distress/mental health concerns
2. The current religious holiday policy needs to be revised with clear guidelines for requesting off a religious holiday
3. The current religious holiday policy is fair towards students of minority religions
4. A new religious holiday policy would create a more inclusive environment
5. A new religious holiday policy that would not use wellness days would positively impact my mental health
6. It is important to me that institutions support my religious beliefs by allowing me time off for my holidays

Please use the below space to share anything with us you feel is important to your experiences with the current religious holiday policy:

________________________
